# Paraneoplastic Pemphigus in a Patient With Relapsed Breast Cancer: An Uncommon Presentation

**DOI:** 10.7759/cureus.99106

**Published:** 2025-12-13

**Authors:** Carlos Salgado, Ivan Cabo, Guilherme Fontinha, Rui Almeida, João Mendes Abreu

**Affiliations:** 1 Stomatology Service - Head, Neck and Skin Surgery, Coimbra Local Health Unit, Coimbra, PRT; 2 Medicine, University of Coimbra, Coimbra, PRT; 3 Medicine, Clinical and Academic Centre of Coimbra, Coimbra, PRT; 4 Pathology, Coimbra Local Health Unit, Coimbra, PRT; 5 Faculty of Medicine, University of Coimbra, Portugal, PRT

**Keywords:** autoimmune diseases, breast neoplasms, neoplasm recurrence, paraneoplastic pemphigus, vesiculobullous diseases

## Abstract

Paraneoplastic pemphigus (PNP) is a rare and severe autoimmune mucocutaneous blistering disorder most commonly associated with hematologic malignancies. Its occurrence in solid tumors, particularly breast cancer, is exceedingly uncommon. PNP may precede, coincide with, or signal recurrence of an underlying neoplasm.

We report the case of a 65-year-old woman presenting with a six-month history of recurrent, painless palatal blisters. Clinical examination revealed multiple intraoral vesicles and a subtly positive Nikolsky sign. Her medical history was notable for breast cancer treated 11 years prior, with oncology discharge in 2021. Biopsy with histopathology and direct immunofluorescence confirmed intraepithelial blistering with IgG deposition, consistent with PNP. Positron Emission Tomography - Computed Tomography (PET-CT) demonstrated widespread recurrence of breast cancer with nodal, hepatic, adrenal, and osseous involvement. The oral lesions were managed successfully with low-dose topical corticosteroids, while systemic treatment for metastatic breast cancer included chemotherapy and palliative radiotherapy.

This case highlights an unusual presentation of PNP secondary to recurrent breast cancer more than a decade after initial treatment. The subtle but persistent mucosal lesions served as an early clinical clue to underlying malignancy progression. Prompt recognition and biopsy-based confirmation were essential to establishing the diagnosis and guiding oncologic management.

PNP should be considered in the differential diagnosis of chronic, unexplained oral vesiculobullous lesions, even in patients with remote histories of solid tumors. Heightened clinical suspicion may facilitate earlier detection of malignancy recurrence.

## Introduction

Pemphigus is a group of autoimmune disorders that are characterized by the disruption of adhesion between keratinocytes at the desmosomal level [1,2]. This process ultimately results in the formation of intraepithelial blisters in the skin, mucous membranes, or both, which can be identified through a specific clinical manifestation known as the Nikolski sign [2-4].

Although pemphigus vulgaris is the most common presentation, other pemphigus variants are known to cause oral vesicobullous lesions. The paraneoplastic variant is reported in fewer than 500 cases in the literature and is associated with a poor prognosis, with a median survival time of 24 months [4,5].

It has been observed that pemphigus-induced paraneoplastic oral lesions tend to appear concurrently with the primary neoplastic manifestations. However, these lesions have also been documented during recurrences. Notably, in approximately 30 to 45% of cases, the manifestation of these symptoms occurs before the diagnosis of an underlying cancer [6]. The pathogenesis of this condition is not fully understood, but it is hypothesized that the underlying malignancy might trigger an immunological dysregulation, culminating in the production of autoantibodies that target the adhesion proteins on the skin and mucous membranes [7].

Non-hematologic neoplasms represent the minority of cases, comprising between 12% [7] and 16% [6] of all paraneoplastic pemphigus (PNP), with adenocarcinoma responsible for about half of those [6]. Therefore, the presence of PNP lesions is an infrequent consequence of breast cancer [8].

The purpose of this article is to report a highly uncommon instance of PNP in a patient with a recurrence of breast cancer after a period of 11 years since the initial diagnosis.

## Case presentation

**Figure 1:**
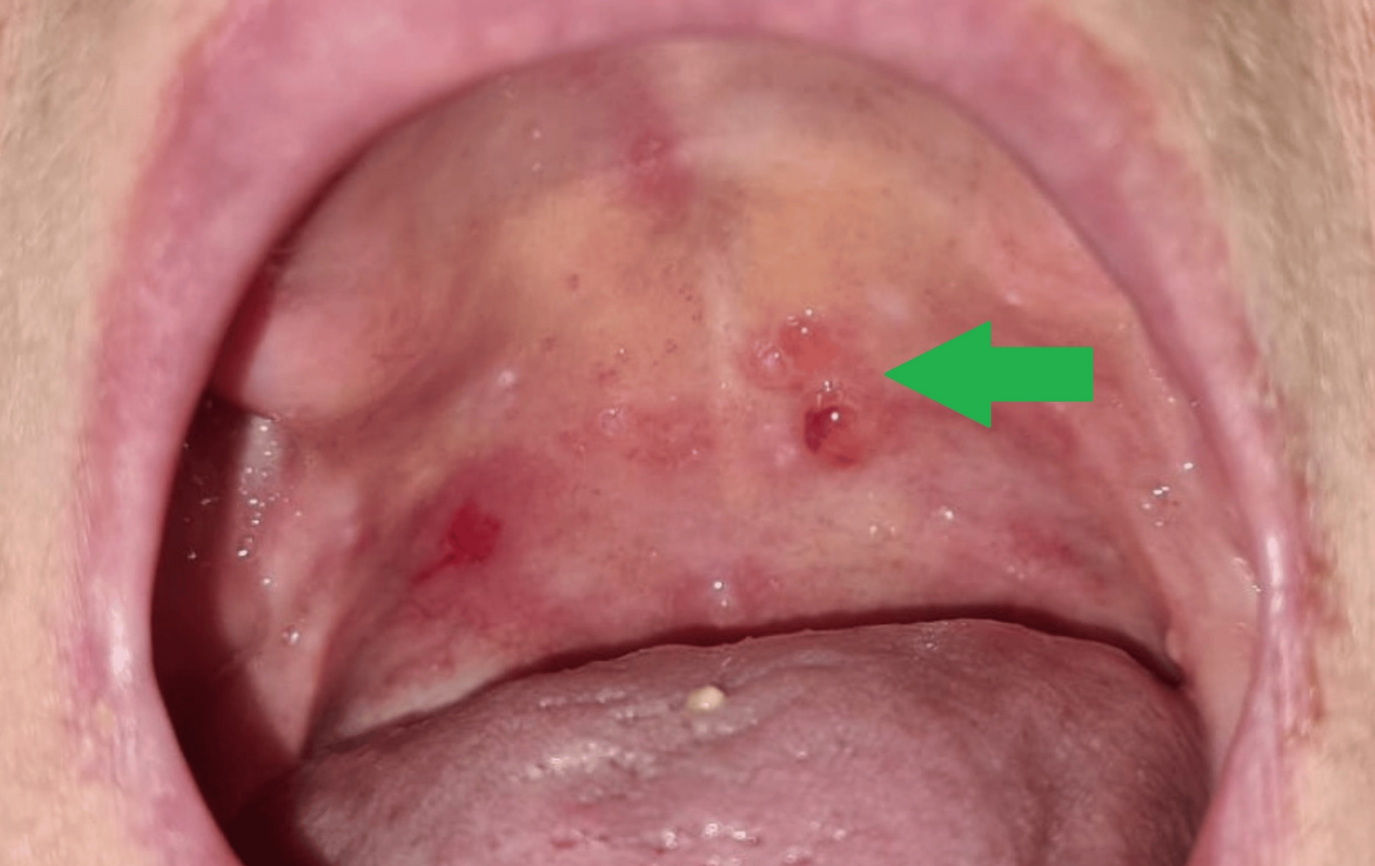
Oral Vesiculobullous Lesions

A 65-year-old woman was admitted to the emergency department due to worsening discomfort associated with the presence of palatal blisters, characterized by at least a six-month cyclical pattern, fluctuating between periods of exacerbation and improvement. The patient's medical history included a prior diagnosis of right breast cancer 11 years prior, staged as pT1bN0, and managed with a tumorectomy and accompanied by a negative sentinel lymph node biopsy. Subsequent evaluation deemed the patient cancer-free in 2021, resulting in discharge from the oncology department. Intraoral examination revealed five friable, painless blisters on both the soft and hard palate. These blisters contained a clear serous liquid, with the largest measuring up to 5 millimeters in diameter (Figure 1).

In addition, during the extraoral evaluation, multiple enlarged cervical and axillary lymph nodes were palpable, accompanied by pronounced lymphedema in the right arm. After the initial evaluation, a Nikolsky sign assessment was conducted, which yielded a subtle but positive result.

**Figure 2:**
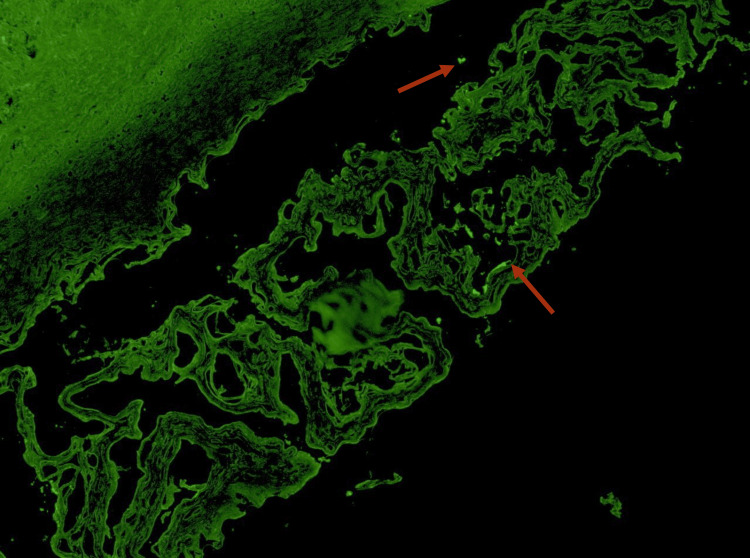
Direct Immunofluorescence with IgG staining IgG deposits (red arrows)

**Figure 3:**
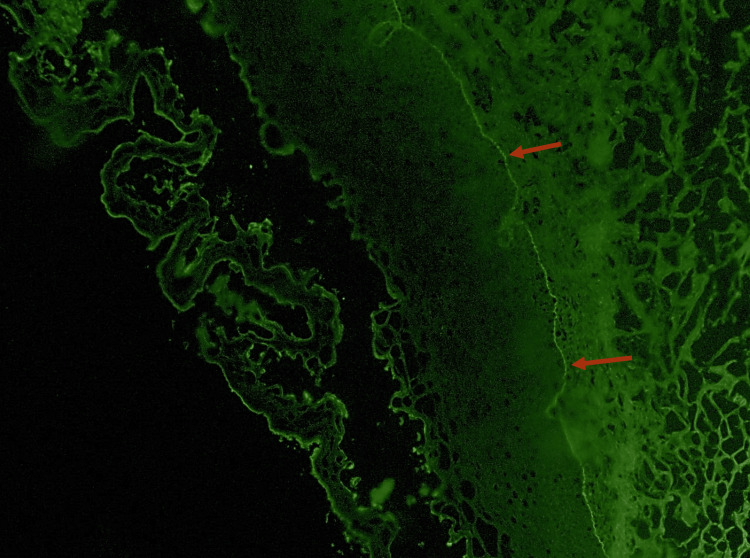
Direct Immunofluorescence with C3 staining C3 deposits (red arrows)

Considering these findings, a differential diagnosis of an oral bullous disease was proposed, namely, vulgar and paraneoplastic pemphigus and mucous membrane pemphigoid. The diagnosis was determined by conducting an incisional biopsy of the buccal mucosa. Two fragments were obtained for subsequent pathology analysis; one specimen was preserved in formaldehyde, while the other was kept fresh to be submitted to direct immunofluorescence examination. This examination facilitated the identification of intraepithelial blisters that exhibited deposition of IgG (Figure 2) and C3 (Figure 3) in the basal layer beneath.

**Figure 4:**
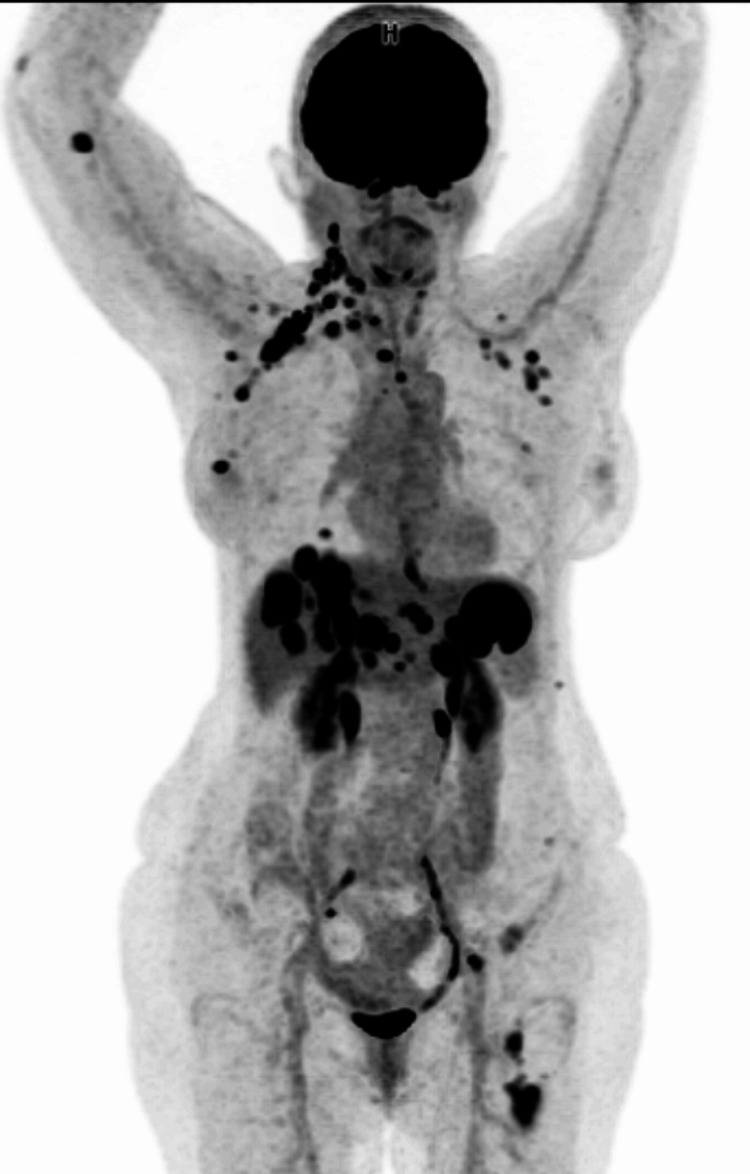
Patient's PET-CT Scan The exam shows multiple scattered adenopathies and hyperfunctioning masses in the right breast, liver, left adrenal gland, and bone

In addition, the presence of lymph nodes and the exacerbation of lymphedema prompted the request for a PET-CT scan, which revealed multiple scattered adenopathies and hyperfunctioning masses in the right breast, liver, left adrenal gland, and bone (Figure 4).

**Figure 5:**
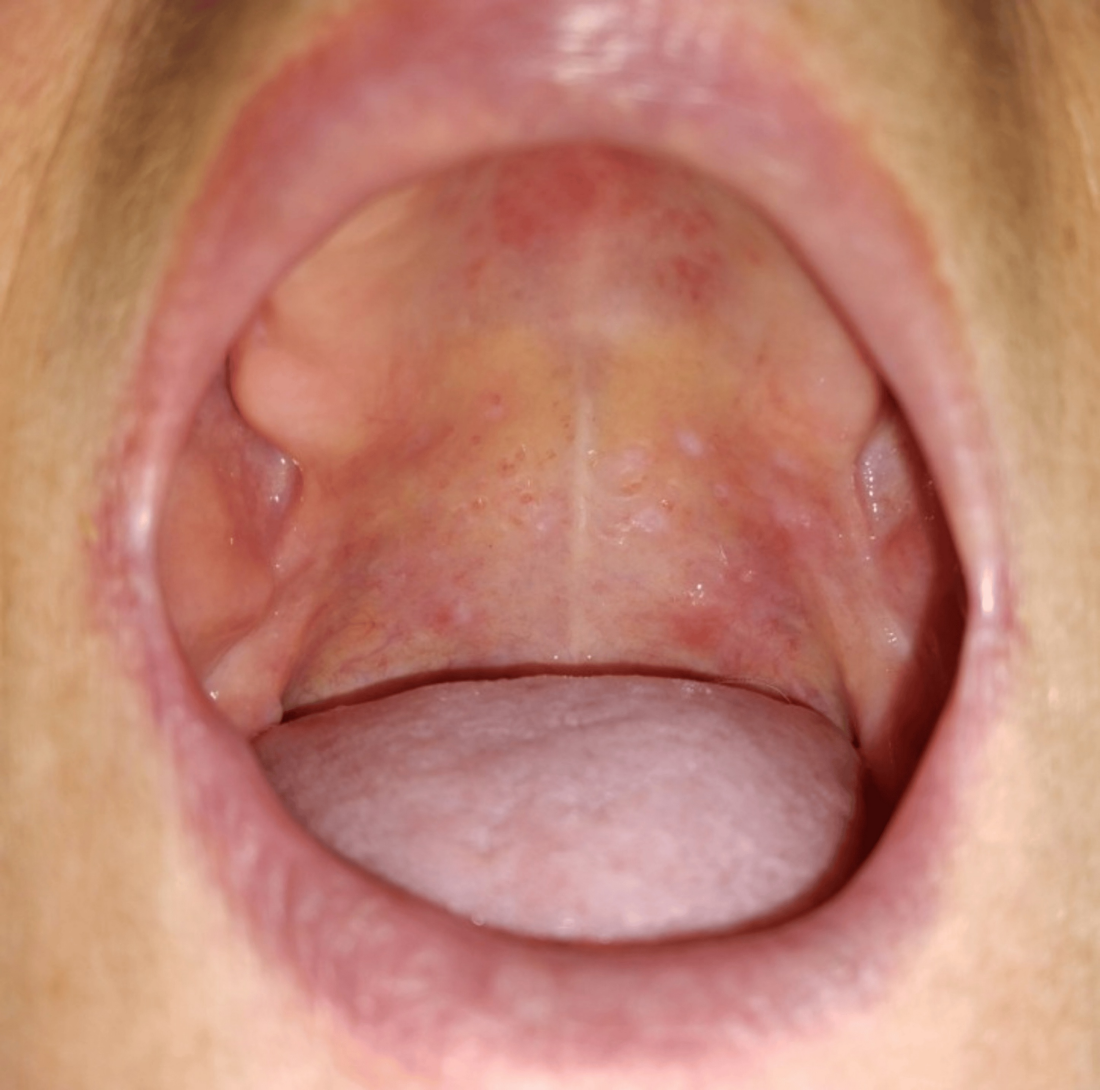
Normal Palatal Mucosa

This finding validated our hypothesis concerning the diagnosis of paraneoplastic pemphigus in instances of both local cancer recurrence and distant metastasis. Following, the oral lesions were successfully treated with the topical application of 1ml of a 0.5mg/ml solution of betamethasone four times a day (Figure 5).

Regarding the patient's breast cancer relapse and metastasis, she was referred to the departments of Gynecology and Radiation Oncology for treatment. This treatment consisted of systemic chemotherapy and palliative radiotherapy. The patient's prognosis is considered reserved.

## Discussion

PNP is an autoimmune condition associated with a neoplasm, most commonly lymphomas. In approximately 30 to 45% of cases, it can present as the initial manifestation of an underlying disease [6].

Its oral variant is characterized by intraoral blisters of varying severity [1,2,7,9], such as what our patient presented. In this case, the initial differential diagnosis included the main etiologies of oral vesiculobullous disease, namely pemphigus vulgaris, paraneoplastic pemphigus, and mucous membrane pemphigoid. Clinically, all three conditions may present with intraoral blistering and a positive Nikolsky sign, making distinction based solely on examination challenging. However, the absence of cutaneous lesions, the chronic relapsing pattern, and the patient’s oncologic history raised early suspicion for a paraneoplastic process. Definitive differentiation was achieved through histopathology and direct immunofluorescence, which demonstrated intraepithelial acantholysis with IgG and C3 deposition, thereby excluding pemphigoid and supporting a diagnosis of PNP [2,9].

The treatment of PNP was initiated with the implementation of low-dose topical corticosteroids, as the patient reported no pain related to the blisters, only a slight discomfort during meals [2,9]. The patient also received systemic chemotherapy for breast cancer, comprising six cycles of docetaxel and stereotactic radiotherapy for bone metastasis [10-12]. Presently, the patient exhibits no signs of pemphigus lesions and maintains a stable disease state, although the reserved prognosis.

However, this may not always be the evolution of these cases. Maglie et al. [8] and Ferguson et al. [13] describe clinical cases of patients developing PNP amid the onset of breast cancer, but both authors report difficulties in the management of the blistering lesions. In their reports, they describe that the PNP lesions were only successfully treated after the malignancy had been treated.

This underscores the significance of a multidisciplinary approach to patients with complex conditions, aiming to enhance both their overall survival and quality of life, particularly when confronted with a condition associated with a poor prognosis.

## Conclusions

Paraneoplastic pemphigus poses a significant diagnostic challenge due to its rarity, variable mucocutaneous presentation, and frequent overlap with other autoimmune blistering disorders. This case underscores the notion that subtle, recurrent oral lesions may serve as an early clinical indicator of underlying malignancy, including late recurrence of solid tumors such as breast cancer.

The patient's presentation, more than a decade after initial tumor treatment, underscores the necessity of maintaining a high level of vigilance when evaluating chronic vesiculobullous manifestations, particularly in individuals with a history of neoplasia. Early recognition, prompt biopsy with immunofluorescence, and coordinated interdisciplinary management are essential to establishing the diagnosis, initiating appropriate treatment, and identifying the associated malignancy.

This case demonstrates the critical importance of a comprehensive clinical evaluation in identifying rare paraneoplastic conditions. It underscores that even minor or asymptomatic oral lesions require meticulous investigation to prevent delays in recognizing serious systemic diseases.
